# Whole genome sequencing of an African American family highlights toll like receptor 6 variants in Kawasaki disease susceptibility

**DOI:** 10.1371/journal.pone.0170977

**Published:** 2017-02-02

**Authors:** Jihoon Kim, Chisato Shimizu, Stephen F. Kingsmore, Narayanan Veeraraghavan, Eric Levy, Andre M. Ribeiro dos Santos, Hai Yang, Jay Flatley, Long Truong Hoang, Martin L. Hibberd, Adriana H. Tremoulet, Olivier Harismendy, Lucila Ohno-Machado, Jane C. Burns

**Affiliations:** 1 Department of Biomedical Informatics, University of California San Diego, La Jolla, California, United States of America; 2 Department of Pediatrics, University of California San Diego, La Jolla, California, United States of America; 3 Rady Children’s Institute for Genomic Medicine, Rady Children’s Hospital, San Diego, California, United States of America; 4 Bioinformatics and Systems Biology Graduate Program, University of California San Diego, La Jolla, California, United States of America; 5 Illumina, San Diego, California, United States of America; 6 Genome Institute of Singapore, Singapore, Singapore; 7 Depatment of Pathogen Molecular Biology, London School of Hygiene and Tropical Medicine, London, United Kingdom; 8 Rady Children’s Hospital San Diego, San Diego, California, United States of America; 9 Moores Cancer Center, University of California San Diego, La Jolla, California, United States of America; National Cancer Institute, UNITED STATES

## Abstract

Kawasaki disease (KD) is the most common acquired pediatric heart disease. We analyzed Whole Genome Sequences (WGS) from a 6-member African American family in which KD affected two of four children. We sought rare, potentially causative genotypes by sequentially applying the following WGS filters: sequence quality scores, inheritance model (recessive homozygous and compound heterozygous), predicted deleteriousness, allele frequency, genes in KD-associated pathways or with significant associations in published KD genome-wide association studies (GWAS), and with differential expression in KD blood transcriptomes. Biologically plausible genotypes were identified in twelve variants in six genes in the two affected children. The affected siblings were compound heterozygous for the rare variants p.Leu194Pro and p.Arg247Lys in Toll-like receptor 6 (*TLR6*), which affect TLR6 signaling. The affected children were also homozygous for three common, linked (r^2^ = 1) intronic single nucleotide variants (SNVs) in *TLR6* (rs56245262, rs56083757 and rs7669329), that have previously shown association with KD in cohorts of European descent. Using transcriptome data from pre-treatment whole blood of KD subjects (n = 146), expression quantitative trait loci (eQTL) analyses were performed. Subjects homozygous for the intronic risk allele (A allele of *TLR6* rs56245262) had differential expression of Interleukin-6 (IL-6) as a function of genotype (p = 0.0007) and a higher erythrocyte sedimentation rate at diagnosis. TLR6 plays an important role in pathogen-associated molecular pattern recognition, and sequence variations may affect binding affinities that in turn influence KD susceptibility. This integrative genomic approach illustrates how the analysis of WGS in multiplex families with a complex genetic disease allows examination of both the common disease–common variant and common disease–rare variant hypotheses.

## Introduction

Although the ability to generate whole genome sequences (WGS) from individual subjects has existed for over a decade, the use of such methods to discover novel disease-causing variants in multiplexed families affected by complex genetic disease has been limited [1]. However, robust methods have been developed to identify rare, disease causative variants in WGS of families with monogenic diseases [2, 3]. Furthermore, such methods have proven useful in identifying individual patients with common diseases that are caused by rare, highly penetrant variants [4]. Individuals with rare Mendelian forms of common complex diseases are often distinguished by extreme phenotypes–such as very early onset, or disease refractory to usual treatments–and by multiple affected members in single families [5]. Here we sought to test this hypothesis in a multiplexed family with Kawasaki disease (KD).

Susceptibility to KD, the most common cause of acquired heart disease in children, is postulated to result from a complex set of genetic variants of which only a limited number have been validated to date [6]. This self-limited illness of unknown etiology presents with the sudden onset of fever and mucocutaneous signs and is associated with coronary artery vasculitis. Inflammation in the arterial wall can compromise the structural integrity, which leads to aneurysm formation in 25% of untreated children [7]. The major sequelae of aneurysms include thrombosis, scarring with stenosis, myocardial ischemia, infarction, and death [8–11]. KD is over-represented among children of Asian descent. In Japan, the country of highest incidence (306/100,000 children <5 years; one in every 60 male and 75 female children affected, respectively), there are more than 14,000 new cases each year and rates continue to rise [12]. In the United States, system dynamic models suggest that by 2030, one in every 1600 adults in the U.S. will have suffered from KD [13]. Data from limited patient series suggests that African American is disproportionately affected by KD [14–16]. Despite their apparent increased susceptibility, children of African American descent has been excluded from previous KD genetic analyses. As with other complex disorders, elucidation of the genetic determinants of KD has hitherto relied on candidate gene and genome-wide association studies (GWAS) using matched population controls and family linkage studies [17–27]. Thus far, many of the associated genetic variants have been located in introns with no associated molecular function identified [6]. Only the C allele of SNV rs28493229 in the inositol 1,4,5-trisphosphate 3-kinase C (*ITPKC*) gene on chromosome 19q13.2 has been shown to affect gene transcription and impact intracellular calcium signaling and inflammasome activation [22, 28].

Here, we examined the common disease–rare variant hypothesis in KD in an African Americans family with two affected and two unaffected siblings and their unaffected, biologic parents. We report rare, likely pathogenic genotypes in biologically plausible genes that co-segregated with disease in whole genome sequences.

## Results

**Fig 1:**
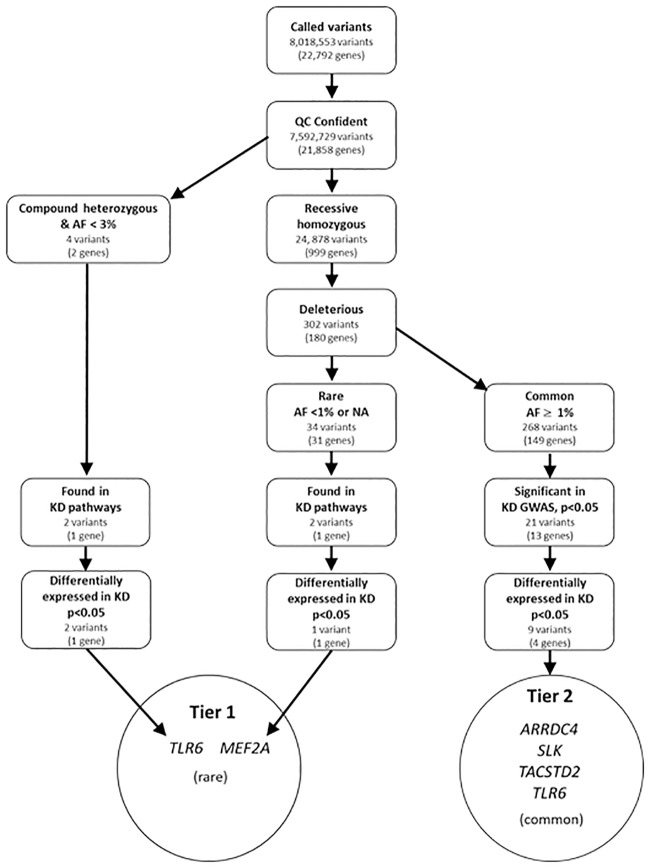
Workflow of variant discovery and validation. The diagram illustrates a discovery and validation workflow starting from called variants in an African American family of six members with two KD-affected children as a discovery analysis on WGS followed by knowledge-based filtering derived from published GWAS, disease pathogenesis, and gene-level validation using differential transcript abundance. Confidence filter: a call quality at least 20 and read depth at least 10. Differentially expressed: ≥ 1.2-fold change between acute and convalescent whole blood transcripts. Abbrv.: AF: allele frequency; NA: not available, GWAS: genome-wide association study; KD: Kawasaki disease; *TLR6*: *Toll-like receptor 6*; *MEF2A*: *myocyte enhancer factor 2A; ARRDC4*:*arrestin domain containing 4*; *SLK*: *STE20 like kinase*; *TACSTD2*: *tumor-associated calcium signal transducer 2*.

The genomes of all six family members were sequenced with paired, short reads to an aligned mean read depth of 33.2-fold. Unique nucleotide variants (8,018,553) were identified in the family, of which 7,592,729 were of high quality (Fig 1). We created three filter pipelines for further analysis. First, we applied the following filters: recessive homozygous only in the affected children, potentially deleterious (located in an exon, promoter region, splice site, or 3’UTR), and rare (allele frequency <1% or not available in 1,000 Genomes database). To these 34 variants in 31 genes, we applied two additional filters: gene found in KD pathway (defined by Ingenuity Pathway Analysis) and differentially expressed (p<0.05) in our KD transcriptome database [29]. This identified a CAG repeat variant (CAG_10_ homozygosity in the two affected siblings) in *myocyte enhancer factor 2A* (*MEF2A)*. The reference allele for this variant is CAG_11_ and the variant was predicted to be deleterious. The deletion was confirmed by resequencing all six family members (S1 Table).

**Fig 2:**
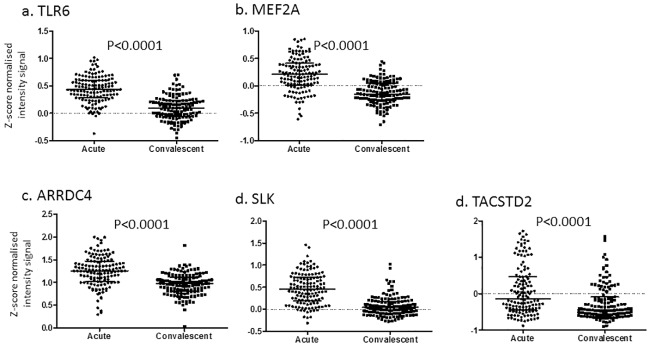
Paired acute and convalescent whole blood gene expression levels for 131 KD subjects for the six genes implicated in KD susceptibility. P values are uncorrected. Transcript levels from microarray database as previously described, not corrected for cell number [29]. Abbrv.: *TLR6*: *Toll-like receptor 6; MEF2A*: *myocyte enhancer factor 2A; ARRDC4*:*arrestin domain containing 4*; *SLK*: *STE20 like kinase*; *TACSTD2*: *tumor-associated calcium signal transducer 2*.

An alternative set of filters was applied to the 7,592,729 high quality variants: compound heterozygous in two affected siblings only, allele frequency <3% in 1000 Genomes database, found in KD pathway, and differentially expressed in the KD transcriptome database. This resulted in only two rare variants (p.Leu194Pro and p.Arg247Lys) in a single gene (*TLR6*) that were compound heterozygous only in the affected siblings (Table 1). The *MEF2A* and *TLR6* rare and potentially pathogenic variants that were found in KD pathways were grouped together in Tier 1 (Fig 1 and Table 1). Both *TLR6* and *MEF2A* were differentially expressed in acute and convalescent whole blood RNA samples, with the highest expression levels in the acute phase of KD (Fig 2a and 2b).

**Table 1:** Genetic variants found in Tiers 1 and 2.

Tier	Mode of inherit	Gene Symbol	Chr.	rs ID	Gene Region	Risk allele	Allelefreq.*	GWAS p-value
1	CH	*TLR6*	4	35220466	Exonic	C	0.6	NA
1	CH	*TLR6*	4	5743809	Exonic	A	1.5	NA
1	HR	*MEF2A*	15	373652230	Exonic	CAG deletion	NA	NA
2	HR	*TACSTD2*	1	14008	Exonic	T	15.7	0.01
2	HR	*TLR6*	4	12650224	3'UTR	A	21.3	0.002
2	HR	*TLR6*	4	6822503	3'UTR	A	63.3	0.008
2	HR	*TLR6*	4	12645200	3'UTR	T	63.3	0.008
2	HR	*TLR6*	4	5743826	3'UTR	T	4.2	0.003
2	HR	*TLR6*	4	6837101	Promoter	A	38.4	0.03
2	HR	*SLK*	10	6584583	Promoter	C	83.1	0.01
2	HR	*SLK*	10	10786779	Promoter	G	79.3	0.02
2	HR	*ARRDC4*	15	1552673	Promoter	C	76.2	0.03

RH = Homozygous Recessive, CH = Compound Heterozygous, *TLR6*: *Toll-like receptor 6*, *MEF2A*: *Myocyte Enhancer Factor 2A*, *TACSTD2*: *tumor-associated calcium signal transducer 2*, *SLK*: *STE20 like kinase*, *ARRDC4*: *arrestin domain containing 4*,

* 1000 genome allele frequencies for All, GWAS: European descent GWAS [19], NA: not available

To explore whether common, deleterious variants were also preferentially associated with KD, we analyzed 302 recessive homozygous and deleterious variants in 180 genes. Of these, nine variants in four genes were significantly associated with KD in an imputed European descent, KD GWAS dataset (nominal p <0.05), and were differentially expressed in whole blood during acute versus convalescent KD [19, 29]. We used our European descent GWAS because no published association studies of African Americans KD subjects were available. These nine common variants were grouped in Tier 2 (Fig 1). Interestingly, *TLR6* was one of the four Tier 2 genes, as well as *arrestin domain containing 4* (*ARRDC4*), *STE20 like kinase* (*SLK*), and *tumor-associated calcium signal transducer 2* (*TACSTD2*). The Tier 2 *TLR6* variants were located either in the promoter or 3’UTR and had population allele frequencies greater than 3% in the 1,000 Genomes database (risk allele frequency 4.2–63.3%) (Table 1).

### eQTL analysis of common *TLR6* variants in KD

**Fig 3:**
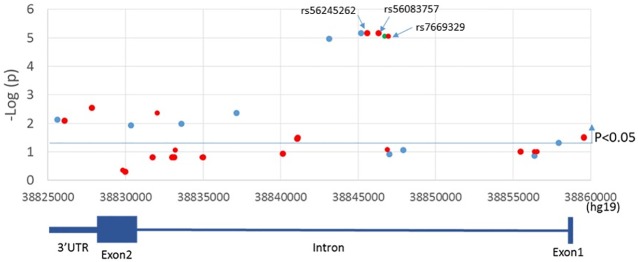
Location of SNVs associated with KD susceptibility in *TLR6*. Association results using the imputed GWAS database were plotted against chromosome location and gene structure of *TLR6*. Red dots show the SNVs for which only the affected children were homozygous recessive. SNVs above the blue line were associated with a P-value <0.05. *TLR6* is encoded on the negative strand so the gene structure is shown 3’ to 5’.

Among the six genes in the two tiers, *TLR6* had multiple variants in both tiers, suggesting that *TLR6* may contribute both to common, sporadic KD as well as uncommon, familial KD. To explore if there were any additional *TLR6* variants for which only the affected children were homozygous, we re-analyzed the original unfiltered *TLR6* WGS from all six family members. We found 34 additional variants for which only the affected children were homozygous (2 synonymous exonic SNVs and 32 intronic SNVs)(S2 Table). All were common variants with allele frequencies of 0.32–0.75 in African datasets in the 1,000 Genomes database. Next, we looked for association of these 34 variants with KD susceptibility in the imputed European descent GWAS dataset [19]. There were three intronic SNVs (rs56245262, rs56083757 and rs7669329) associated with KD susceptibility with p = 6.9x10^-6^ (S2 Table and Fig 3). These three intronic SNVs and the seven SNVs in *TLR6* from Tiers 1 and 2 are shown in Table 2. The two SNVs in the 3’UTR (rs6822503 and rs12645200) were in linkage disequilibrium (LD, with r^2^ = 0.97 in Africans, r^2^ = 1 in European descendants) in the imputed European descent GWAS dataset, as were the three intronic SNVs (rs56245262, rs56083757 and rs7669329), with r^2^ = 1 in both African and European descent cohorts. We chose rs6822503 and rs56245262 (earlier chromosome position) as representative SNVs for additional analysis.

**Table 2:** TLR6 variants in African American family with two affected siblings.

rs ID	Gene Region	Ethnicity				Risk allele	Family members						WGS	GWAS p-value
		AA*	HIS**	ASN	CEU		KD 1	KD 2	Non-KD 1	Non-KD 2	M	F		
12650224	3'UTR	0.23	0.17	0.28	0.03	A	Hom	Hom	Het	-	Het	Het	Tier2	0.002
6822503	3'UTR	0.77	0.46	0.76	0.52	A	Hom	Hom	Het	-	Het	Het	Tier2	0.008
12645200	3'UTR	0.77	0.45	0.76	0.52	T	Hom	Hom	Het	-	Het	Het	Tier2	0.008
5743826	3'UTR	0.08	0.02	0	0.01	T	Hom	Hom	Het	-	Het	Het	Tier2	0.003
35220466	Exon (p.Arg247Lys)	0.01	0	0	0	T	Het	Het	Het	-	-	Het	Tier 1	ND
5743809	Exon (p.Leu194Pro)	0.08	0	0	0	G	Het	Het	-	-	Het	-	Tier 1	ND
56245262	Intronic	0.42	0.37	0.62	0.23	A	Hom	Hom	Het	-	Het	Het	NA	6.9E-06
56083757	Intronic	0.42	0.37	0.62	0.23	G	Hom	Hom	Het	-	Het	Het	NA	6.9E-06
7669329	Intronic	0.42	0.37	0.62	0.23	C	Hom	Hom	Het	-	Het	Het	NA	6.9E-06
6837101	Promotor	0.45	0.34	0.35	0.29	A	Hom	Hom	Het	-	Het	Het	Tier2	0.03

AA: African American, HIS: Hispanic, ASN: Asian, CEU: European descent, KD: Kawasaki disease affected child, Non-KD: unaffected child, M: mother, F: father, GWAS: European descent GWAS [19], Hom: homozygous of risk allele, -: homozygous of non-risk allele, NA: not applicable,

* 1000 Genomes African Ancestry in Southwest US,

** 1000 Genomes:Phase_3:AMR

**Fig 4:**
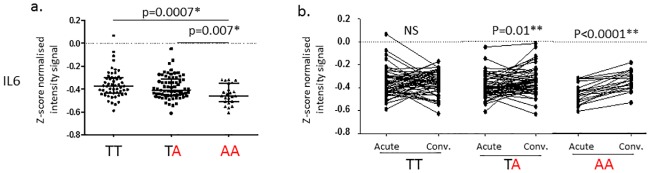
Transcript levels for IL6 in whole blood samples from KD subjects as a function of genotype for rs56245262 in *TLR6*. *TLR6* rs56245262 risk allele (A) is shown in red. Transcriptome data from a. Acute, pre-treatment (n = 146: T/T n = 54, T/A n = 69, A/A n = 23), b. Paired acute, pre-treatment and convalescent (n = 131: T/T n = 53, T/A n = 58, A/A n = 20). P-values were calculated using Mann Whitney test for (a) and Wilcoxon matched-paired test for (b).

To understand the possible effects of common variants in *TLR6* in KD pathogenesis, we performed an eQTL analysis for the two representative SNVs (rs56245262 and rs6822503) and one SNV (rs6837101) in the promoter region of TLR6. Since TLR2/6 activates the transcription factors, NFKB and AP1, we focused the eQTL analysis using only the 415 genes targeted by these transcription factors. For the intronic variant (rs56245262), only one gene (*IL6)* among 415 NFKB and/or AP1 targets showed a significant difference in acute whole blood transcript levels as a function of genotype (nominal p< 0.001). *IL6* transcript levels were lower in subjects homozygous for the risk allele (p = 0.0007 vs. non-risk allele homozygotes, and p = 0.007 vs. heterozygotes) (Fig 4). For variants in the 3’UTR (rs6822503), no gene showed significantly different transcript levels as a function of genotype with a p< 0.001. No genes were regulated as a function of the genotype of the promoter SNV (rs6837101).

### Analysis of patient characteristics as a function of genotype

Since the *TLR6* intronic SNVs were associated with differential expression of IL6, we reasoned that patient clinical characteristics related to inflammation might also vary as a function of genotype. We used our published dataset of 161 subjects [30] with detailed demographic and clinical information and 7,602,343 imputed genotypes (from the Illumina HumanOmni1-Quad^®^ chip) to analyze differences in clinical parameters as a function of genotype for the intronic variant (rs56245262)(Table 3). Homozygosity for the risk allele was more common among self-declared Asians (10 of 24 (44%) A/A genotype). This result was consistent with the observation that the A allele frequency is higher in Asian populations (A allele frequencies in 1000 Genome database: Asian 0.62, A/A 0.42, Hispanic 0.37, European descent 0.23). Of interest, the pre-treatment erythrocyte sedimentation rate (ESR) was higher in subjects homozygous for the risk allele (A/A) (median ESR 80 mm/h for A/A vs. 59 mm/h for T/T, p = 0.01).

**Table 3:** Characteristics of subjects (n = 161) by genotype of TLR6 rs56245262.

	TLR6 rs56245262			p*
	A/A n = 24	A/T n = 78	T/T n = 59	
Male, n (%)	15 (63)	48 (62)	33 (56)	NS
Age, years median (IQR)	2.7 (0.9–3.8)	2.6 (1.4–4.1)	2.2 (1.2–4.4)	NS
Illness Day, median (range)**	6 (3–9)	6 (3–10)	6 (2–10)	NS
Ethnicity, n (%)				
Asian	10 (44)	13 (17)	5 (8)	0.002***
African-American	1 (0)	5 (6)	0 (0)	NS
Caucasian	3 (12)	18 (23)	21 (36)	NS
Hispanic	3 (24)	22 (28)	17 (29)	NS
More than race	7 (20)	20 (26)	16 (27)	NS
Coronary artery status				
Aneurysms, n (%)	3 (13)	7 (9)	8 (14)	NS
Dilated, n (%)	4 (16)	19 (24)	13 (22)	
Normal, n (%)	17 (71)	52 (67)	38 (64)	
Z-worst, median (IQR)	1.5 (1.0–2.6)	1.7 (1.1–2.9)	1.9 (1.3–2.9)	NS
IVIG resistant, n (%)	4 (17)	19 (25)	12 (20)	NS
Lab data, median (IQR)				
WBC, ×10^3^/mm^3^	16.3 (11.6–19.4)	13.2 (10.5–18)	13.9 (11.7–18.7)	NS
Absolute neutrophil, ×10^3^/mm^3^	10.7 (7.4–15.2)	9.0 (6.7–12.5)	8.4 (6.4–11.6)	NS
Platelet count, ×10^3^/mm^3^	401 (334–462)	400 (292–500)	392 (312–472)	NS
ESR, mm/h	80 (57–105)	59 (41–80)	59 (44–78)	0.01****
CRP, mg/dl	7.9 (4.3–13.6)	8.4 (4.7–16.8)	8.2 (5.2–16.1)	NS
GGT	34 (25–96)	48 (18–134)	41 (22–101)	NS

* p-values were calculated by Mann–Whitney U test for continuous variables and chi test for categorical variables except ethnicity comparison.

** Illness day 1: first calendar day of fever.

*** Fisher-Freeman-Halton test. Asian comparing to all other ethnicities combined.

**** Mann-Whitney test for the levels of A/A vs. T/T genotype. IVIG: Intra venous immunoglobulin G therapy CRP: C-reactive protein, ESR: Erythrocyte sedimentation rate, WBC: White blood count

## Discussion

Analysis of WGS of an African Americans family with two affected and two unaffected siblings and their unaffected, biologic parents highlighted genetic variation in *TLR6* in KD susceptibility. These *TLR6* variants included both compound heterozygosity for two rare, likely deleterious SNVs and homozygosity for common KD risk SNVs. Subsequently, using an acute KD whole blood transcriptome data set, eQTL analysis of the common SNVs suggested decreased transcript levels of *IL6* and higher ESR at diagnosis in individuals homozygous for the risk allele. This integrative genomic approach illustrates how WGS in families with multiple members affected with a complex genetic disease can yield insights into both the common disease–common variant and common disease–rare variant hypotheses.

### TLR6- NFKB signaling pathway

TLRs recognize pathogen-associated molecular patterns (PAMPS), lead to activation of the transcription factors, NFKB and AP1, and transcription of genes that control inflammation. TLR6 forms a heterodimer with TLR2 that recognizes peptidoglycan, diacyl lipoproteins, and zymosan derived from Gram positive bacteria, mycoplasmas, and fungi, respectively [31]. TLR6 is constitutively expressed in humans on myeloid dendritic cells, monocytes, B cells, coronary artery endothelial cells (EC), and coronary artery vascular smooth muscle cells [32, 33]. In contrast, TLR2 is widely expressed on immune cells but only expressed on human EC and vascular smooth muscle cells following induction by pro-inflammatory cytokines. Population differences in downstream cytokine production have been observed following TLR stimulation. In a study of African children, higher levels of TNFα were produced following *in vitro* stimulation with a specific TLR2/6 ligand in a whole blood assay when compared to children of European descent [34].

We found 41 *TLR6* variants located in the promoter, intron 1, exon 2, and the 3’UTR associated with KD susceptibility. The three common intronic SNVs (rs56245262, rs56083757 and rs7669329) influenced the transcription of *IL6* and were associated with KD susceptibility both in the European descent GWAS and the African Americans family. IL6, a key cytokine controlled by the transcription factors NFKB and AP1 among others, is reported to be high in the serum of acute KD patients [35]. Our transcriptome data show that IL6 transcripts are low in risk allele carriers during the acute disease (Fig 4) [29]. The variants were located in a 1.5kb region in the middle of the single 28kb intron, 11kb from the splice donor site and 17kb from the splice acceptor site. Five transcripts of *TLR6* have been predicted to result from alternative splicing (https://www.ncbi.nlm.nih.gov/nuccore?LinkName=gene_nuccore_refseqrna&from_uid=10333) and it is possible that the intronic SNVs could influence splicing efficiency of these variants (S1 Fig). The eQTL database, GTEx (http://www.gtexportal.org/home/), showed that there were tissue-specific effects of these intronic SNVs with the risk allele decreasing *TLR6* transcripts in transformed fibroblasts but increasing *TLR6* transcripts in a transformed B-cell line (S2 Fig). No data were available for other tissues of potential interest in KD including endothelial cells, vascular smooth muscle cells, and cardiomyocytes.

Two of the four *TLR6* 3’UTR SNVs (rs12645200 and rs6822503) were in LD. We failed to find any microRNAs that were predicted to bind these four loci according to the miRdSNV data base (http://mirdSNV.ccr.buffalo.edu/index.php [36] and miRNASNV V2 (http://bioinfo.life.hust.edu.cn/miRNASNV2/index.php) [37, 38]. For one of the 3’UTR variants, rs6822503, the risk allele was in weak LD (r2 = 0.58, D’ = 0.94) with the C allele of the exonic variant c.G1083C (rs3821985) in Africans, and the two affected siblings were homozygous for the C allele at this locus (S1 Table). Shey et al stimulated whole blood from 70 healthy Africans with the diacylated lipopeptides, FSL-1 and PAM2 (TLR6/2 ligands), and found reduced IL6 levels in cells from subjects homozygous for the C allele of rs3821985 compared to G homozygotes [39]. This finding suggests that our KD-affected siblings might produce lower levels of IL6 upon TLR2/6 stimulation.

**Fig 5:**
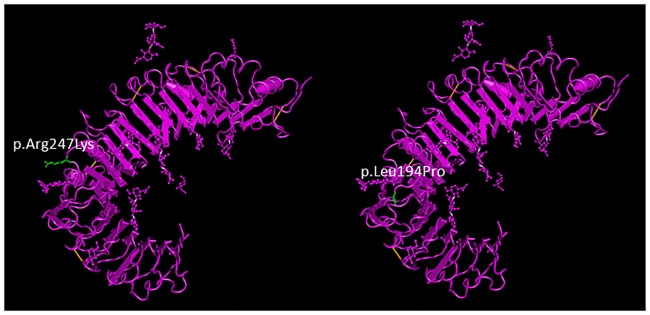
TLR6 exonic SNV positions. Positions of the exonic SNVs rs5743809 and rs3522046 on the TLR6 protein diagrammed using NCBI Molecular Modeling database (MMDB). The replaced amino acids (p.Leu194Pro and p.Arg247Lys) are shown in green. Variants are located in the extra-cellular domain in the predicted ligand-binding region and may alter hydrogen binding.

The two affected children were compound heterozygotes for the two SNVs in *TLR6* exon 2 (p.Leu194Pro and p.Arg247Lys) having inherited the former from the mother and the latter from the father. These SNVs change amino acids located on the extracellular surface of TLR6 in a region predicted to be involved with ligand binding, which could influence ligand-binding affinity (Fig 5). 3D proteomic structure modeling of the two non-synonymous mutations was computationally predicted (SNPs3D: http://www.snps3d.org/modules.php?name=SnpAnalysis&locus_ac=10333). p.Leu194Pro is predicted to be deleterious due to loss of an intramolecular hydrogen bond and p.Arg247Lys is a variant classified as non-deleterious but on the protein ectodomain. The functional impact of these *TLR6* variants was tested using an NF-κB luciferase reporter assay in human embryonic kidney 293T cells expressing the *TLR6* variants and stimulated with TLR agonists [40]. p.Arg247Lys showed a 15.6% decrease in ability to respond to the TLR2/6 agonist, PAM2CSK4. Cells transfected with p.Leu194Pro constructs showed a more marked decrease in NF-kB activation (25.4%). Thus, compound heterozygosity for these SNVs in the affected children is expected to reduce NF-kB activation.

### Role of NFKB and IL6 in the modulation of immune responses

A self-limited inflammatory disease like KD must activate potent anti-inflammatory pathways that ultimately lead to the resolution of inflammation. TLR2/6 stimulation initiates inflammation but also stimulates the regulatory compartment of the immune response. In mice, stimulation of TLR2/TLR6 expressed on dendritic cells leads to their differentiation into tolerogenic dendritic cells secreting IL-10 and promotes T cell differentiation into a regulatory (Treg) phenotype [41]. Work by Franco et al. has highlighted the importance of IL-10 secretion by natural regulatory T cells and tolerogenic myeloid dendritic cells in the resolution of inflammation in KD patients [42, 43]. Downstream of TLR2/6, both NFKB and IL6 have complex immune functions [44, 45]. In the carrageenan-induced rat pleurisy model, blocking NFKB led to protracted inflammation with reduced leukocyte apoptosis and decreased release of the anti-inflammatory molecule, TGFβ1, thus highlighting the important role of NFKB in the resolution of inflammation [44].

**Fig 6:**
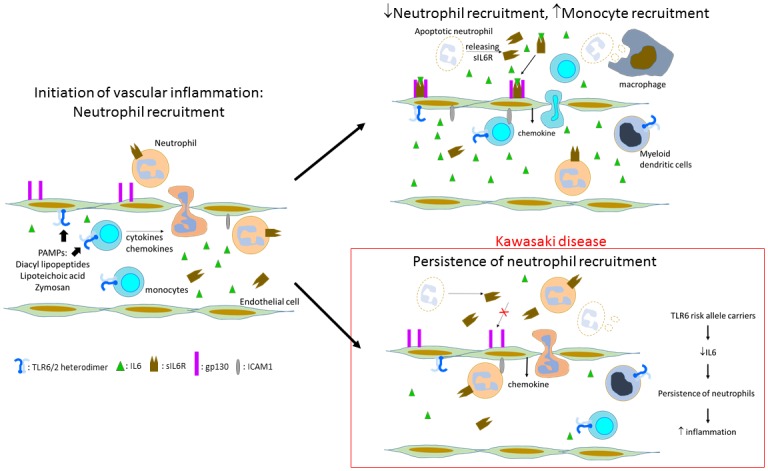
Hypothetical role of decreased IL6 signaling in the persistence of vascular inflammation in acute KD. The *TLR6* variants associated with KD are predicted to yield lower levels of NFKB upon stimulation, thus resulting in lower levels of IL6. During vascular inflammation, neutrophil apoptosis is associated with shedding of the soluble IL6R-IL6 complex, which binds to gp130 on endothelial cells. This stimulates a signaling pathway that switches the adhesion molecule and chemokine profile to one that favors attraction of monocytes to the vessel wall with subsequent downregulation of vascular inflammation. In KD, lower levels of IL6 may lead to persistence of neutrophil recruitment to the vessel wall and prolonged inflammation.

IL6 functions by binding the IL6 receptor (R) and gp130, a transmembrane signal transduction protein. The IL6R is expressed only on hepatocytes and a subset of inflammatory cells including macrophages, neutrophils, and naïve T cells. However, gp130 is ubiquitously expressed [45]. During acute inflammation, neutrophils infiltrate tissues and undergo apoptosis with shedding of IL6-sIL6R. This complex binds gp130 on endothelial cells leading to signal transduction that results in monocyte/macrophage recruitment and removal of apoptotic neutrophils. Thus, reduced levels of IL6 might be expected to allow persistence of neutrophils in the arterial wall and in the circulation. Although IL6 levels in the serum are high in acute KD, this may represent synthesis of IL6 by hepatocytes as part of the acute phase response rather than synthesis by circulating immune cells as eQTL are often tissue-specific [35]. This could have important implications for persistence of the acute inflammatory state in KD (Fig 6). Of interest, a pilot study of tocilizumab (monoclonal antibody against human IL6R) plus IVIG for treatment of acute KD was terminated for safety concerns when the first three patients enrolled developed CAA (Prof. Emeritus Shumpei Yokota, Yokohama City University, Japan, personal communication).

### Tier 1 genes

*TLR6* and the transcription factor, *MEF2A*, were the two Tier 1 genes. They were found in KD pathways and were differentially expressed in the KD transcriptome database with significantly higher levels in the acute phase. *MEF2A* plays a critical role in transcriptional activation of IL-2 during T cell activation [46]. Our associated SNV, rs373652230, was a (CAG) deletion at amino acid 420 in a poly-lysine tract and multiple studies have reported (CAG)_n_ variants associated with coronary artery disease, although the mechanism underlying this association has not been elucidated [47]. Although the Ingenuity Variant Analysis identified the CAG_10_ variant as rare (allele frequency <1%), studies in European descent, Asian, and Turkish populations found allele frequencies from 12.7–21.9%. We were unable to find data regarding the allele frequency of the (CAG) repeats in African Americans populations. Of interest, the intronic *TLR6* SNV, rs56083757 and the promoter SNV rs6837101, were also predicted to influence the DNA binding of MEF2A (HaploReg: http://www.broadinstitute.org/mammals/haploreg/detail_v4.1.php?query=&id=rs56083757). No other functional significance could be assigned to the other SNVs.

The nature of the PAMP that might stimulate TLR6/TLR2 in KD is unknown although a hypothesis has linked KD to inhalation of Candida antigens carried on aerosols arising from agricultural areas in China [48]. Of speculative interest, the glucans from the Candida cell wall are potent ligands for TLR2/6. In addition, C57BL/6 mice are homozygous for the p.Leu194Pro variant in TLR6 and two murine models of coronary artery vasculitis in this species use intraperitoneal injection of either *Candida albicans* or *Lactobacillus casei* cell wall extracts, both of which are potent ligands for TLR2/6[49, 50].

### Tier 2 genes

Six genes harboring nine variants met the criteria of homozygous recessive, predicted deleterious, significantly associated in the imputed GWAS, and differentially expressed in acute versus convalescent KD (Fig 2). The *TLR6* promoter and 3’UTR SNVs were discussed above.

The Tier 2 gene, *TACSTD2*, is involved in calcium signaling and may be linked in this way to KD pathogenesis. Validated calcium signaling genes linked to KD pathogenesis currently include *ITPKC*, *ORAI1*, and *SLC8A1* [22, 30, 51]. *TACSTD2* encodes for Trop2, a membrane-spanning protein that transduces an intracellular calcium signal. The protein is over-expressed in many epithelial cancers and is thought to be involved in metastasis and malignant cell invasion [52]. Its transcription is regulated by a number of transcription factors including NFKB and its expression was high in acute versus convalescent KD.

The serine/threonine Ste-like protein kinase, SLK, belongs to the family of germinal center kinases, is ubitquitously expressed, and is involved with stress-induced apoptosis, cytoskeletal remodeling, and cell motility [53]. ARRDC4 is a member of the α-arrestin family, which, as a class, is involved with fine-tuning cellular responses to cell surface signals [54]. Both *SLK* and *ARRDC4* were transcriptionally upregulated in acute KD but the mechanism through which these genes may participate in KD susceptibility is unclear.

### Strengths and limitations

This is the first analysis of WGS in an African Americans family and provides a database that can be mined for future studies of genetic structure in this population. Many different filters can be applied for subsequent analyses that may uncover additional variants that influence susceptibility to KD. Neither affected child developed coronary artery aneurysms, so only variants affecting susceptibility can be analyzed from this dataset. We recognize that using a whole blood transcriptomic database from a mixed ethnic population could miss transcripts that are only differentially expressed in relevant cardiovascular tissues and among African Americans. This analysis underscores the need to focus genetic and genomic studies on minority populations such as African Americans who are disproportionately affected by KD compared to children of European descent.

### Conclusion

The first analysis of WGS from an African Americans family with two siblings affected with KD revealed genetic variation in *TLR6* that may be linked to the pro-inflammatory state during acute KD. Previous GWAS and linkage association studies had not identified this gene as influencing susceptibility to KD. Another variant, in *TACSTD2*, has an intriguing link to calcium signaling that will need to be pursued in future studies. The analytic approach presented here is a novel method for finding potentially relevant variants in WGS in families with individuals affected by complex genetic diseases.

## Materials and methods

### Subjects

Members of the African American family selected for whole genome sequencing included two affected sons, an older unaffected son and daughter, and unrelated/unaffected father and mother, all of whom provided written informed consent for study participation. The study was approved by the Institutional Review Board at the University of California San Diego. The two affected subjects were both diagnosed by one of the co-authors (JCB). Neither developed coronary artery abnormalities by echocardiogram and both responded to a single dose of IVIG with defervescence and resolution of inflammation.

### Whole genome sequencing

Whole blood samples or Scope^®^ mouthwash rinses were obtained from family members and 10 μg of genomic DNA was extracted and submitted to Illumina Clinical Services Laboratory in San Diego, CA, USA for sequencing using HiSeq 2000. DNA was fragmented and attached to the surface of glass microscope slides. Fluorescently labeled nucleotides were used to sequence the fragments. Laser excitation of the nucleotides was followed by digital imaging. The sequence fragments were assessed for quality scores.

### Sequence processing and variant calling

Raw images were processed by Illumina’s CASAVA version 1.8 pipeline to generate six sample FASTQ files for a downstream analysis. The FASTQ files were aligned to a human reference genome (hg19), PCR duplicates marked and variants called using Edico Genome’s DRAGEN pipeline [55], using default parameters.

### Variant prioritization

VCF files were uploaded to Ingenuity Variant Analysis^™^ (QIAGEN Redwood City, CA) for tertiary analysis. Variant prioritization was performed by sequentially applying filters in 7 steps of confidence, genetics (recessive homozygous and compound heterozygous), predicted deleterious, rare, found in KD pathway, significant in published KD GWAS, and differentially expressed in KD transcriptome database (Fig 1). The *Confidence filter* retained variants with a call quality at least 20, read depth at least 10, and eliminated the top 5% of the most exonically variable genes or 100-bp regions in 1000 Genomes to remove false positive variants such as mucin and olfactory receptor genes. The annotated variants were imported into an in-house MySQL database to perform genetic analysis of two inheritance types, recessive homozygous and compound heterozygous. The *Homozygous filter* required homozygous variants to be present exclusively in the two KD affected children but not in any of the four unaffected individuals. The *Compound heterozygous filter* was constructed based on the following rules [56]:

1. A variant is in a heterozygous state in both of KD affected children.
2. A variant must not occur in a homozygous state in any of the four unaffected individuals, i.e. two siblings and two parents.
3. A variant that is in a heterozygous state in an affected child must be heterozygous in exactly one of the parents but not both.

The *Predicted deleterious filter* followed the guideline classification of American College of Medical Genetics (ACMG) and loss-of-function in terms of frameshift, in-frame in/del, start/stop codon change, missense, or splice site loss all implemented in IVA. The *Rare filter* retained variants whose allele frequency was less than 1% (for recessive homozygosity) or 3% (for compound heterozygosity) in any of three public datasets; 1000 genomes, Exome Aggregation Consortium (ExAC), and NHLBI ESP Exomes. The *Pathway filter* generated by Ingenuity program retained genes implicated in pathways relevant to KD pathogenesis. These were defined using the Ingenuity system with the key terms “susceptibility to KD”, “calcium signaling pathway, coronary artery aneurysm”, and “coronary artery abnormalities”. The *GWAS filter* retained variants that were significantly (nominal P-value < 0.05) associated in 405 KD subjects versus 6,252 normal controls in our previously published KD GWAS dataset genotyped on the Illumina HumMap 550 SNV array [19]. Since GWAS targets common SNVs with an allele frequency > 5%, the *GWAS filter* was applied to variants surviving the *Deleterious filter* in Fig 1. Quality control was performed with missing rate, minor allele frequency (MAF) and Hardy-Weinberg Equilibrium (HWE) cutoff values. The original dataset was expanded through imputation using SHAPEIT2 for phasing and IMPUTE2 for imputation with the most recent versions of 1000 Genomes version 3 and HapMap3 CEU panels (hg18) as reference data. LiftOver tool from UCSC Genome Browser (Kent et al. Genome Res 2002 PMID:12045153) was used to convert genomes coordinates from assembly hg18 to hg19. The new imputation with updated reference panels increased the total number of imputed SNVs to 7,602,343 from 4,545,265. The *Transcriptome filter* set gene-level prioritization by retaining differentially expressed genes between 131 paired acute and convalescent whole blood RNA samples (PAXgene).[29] Transcript levels were measured using the Illumina HumanRef-12 V4 BeadChip with 47,000 probes and quality control and analysis were as described. The cutoff for differential expression was an adjusted P-value < 0.05.

### Validation genotyping

#### MEF2A

The six family members were re-sequenced for the MEF2A (CAG)_n_ variant.

Primers were designed using Primer 3: forward primer (caagtccgaaccgatttcac) and reverse primers (gccaagcacaattggagaat) (product size 247 bp). Fifty nanograms of DNA from each sample was amplified using high-fidelity Taq polymerase (Platinum^®^ *Taq* DNA Polymerase High Fidelity, Life Technologies) for 35 cycles following the manufacturer’s instructions. PCR products were resolved on 2% agarose gels, excised, and purified (QIAquick Gel Extraction Kit, Qiagen). PCR products were sequenced using forward and reverse primers (Eaton Bioscience Inc., San Diego).

### eQTL analysis

Detailed methods were described previously [30]. A total of 673 probes from 415 genes from the database of literature-curated human TF-target interactions for NFKB and AP1 were used for eQTL analysis [57].

### Data sharing

Data is available upon request. Users wishing to access our data should contact us.

## Supporting Information

S1 Fig *TLR6* gene structure, predicted splice variants, and location of SNVs. (TIF)

S2 Fig *TLR6* expression as a function of genotype at the *TLR6* intronic SNV (rs56083757) from the database. Genotype-Tissue Expression (GTEx: http://www.gtexportal.org/home/).
(TIF)

S1 Table MEF2A re-sequencing results. (PDF)

S2 Table Variants in the toll like receptor 6 gene in family members. (PDF)
