# Supplementary figures and images for: Whole genome sequencing of an African American family highlights toll like receptor 6 variants in Kawasaki disease susceptibility
Source: PLoS One. 2017 Feb 2;12(2):e0170977. doi: 10.1371/journal.pone.0170977 (PMC5289527; doi:10.1371/journal.pone.0170977)

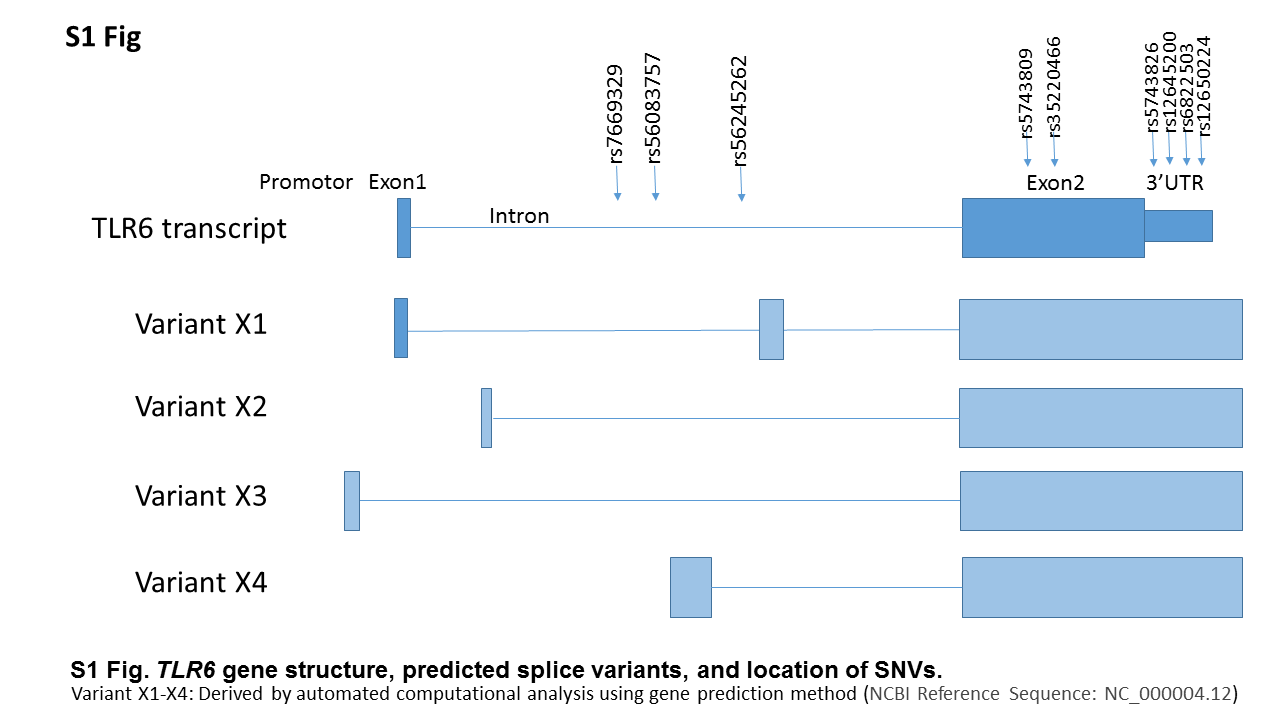

Supplement: S1 Fig — (TIF) [file pone.0170977.s001.tif]

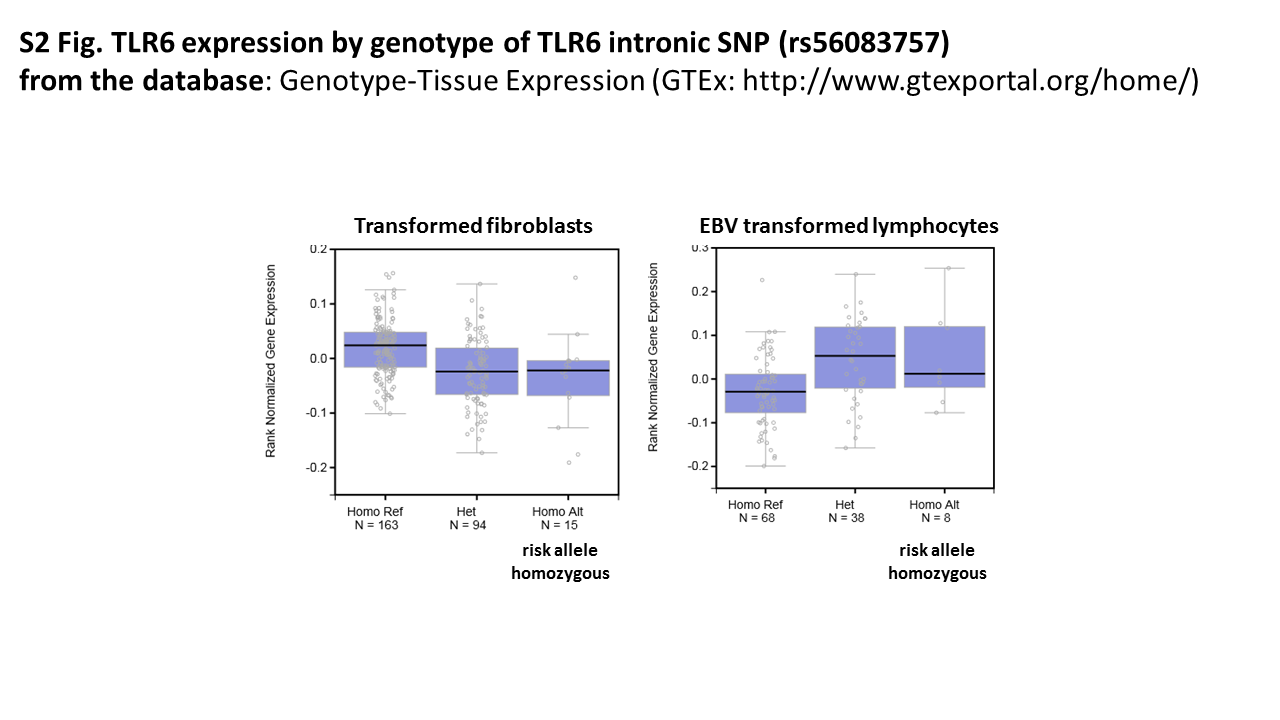

Supplement: S2 Fig — Genotype-Tissue Expression (GTEx: http://www.gtexportal.org/home/). (TIF) [file pone.0170977.s002.tif]
