# Supplementary material for: Whole genome sequencing of an African American family highlights toll like receptor 6 variants in Kawasaki disease susceptibility
Source: PLoS One. 2017 Feb 2;12(2):e0170977. doi: 10.1371/journal.pone.0170977 (PMC5289527; doi:10.1371/journal.pone.0170977)
Supplement: S1 Table — (PDF) [file pone.0170977.s003.pdf]

**S1 Table. MEF2A re-sequencing results**

| **Family member** | **(CTG)n** |
|---|---|
| Affected child 1 | $(CTG)_{10}$/ $(CTG)_{10}$ |
| Affected child 2 | $(CTG)_{10}$/ $(CTG)_{10}$ |
| Father | $(CTG)_{10}$/ $(CTG)_{13}$ |
| Mother | $(CTG)_{10}$/ $(CTG)_{11}$ |
| Unaffected Sibling 1 | $(CTG)_{10}$/ $(CTG)_{11}$ |
| Unaffected Sibling 2 | $(CTG)_{11}$/ $(CTG)_{13}$ |
