# Supplementary material for: Whole genome sequencing of an African American family highlights toll like receptor 6 variants in Kawasaki disease susceptibility
Source: PLoS One. 2017 Feb 2;12(2):e0170977. doi: 10.1371/journal.pone.0170977 (PMC5289527; doi:10.1371/journal.pone.0170977)
Supplement: S2 Table — (PDF) [file pone.0170977.s004.pdf]

S2 Table. Variants in the Toll Like Receptor 6 gene in family members.

dbSNP rs ID	Gene Region	Position	Risk Allele	Genotype of family members						WGS discovery	European descent GWAS p-value	LD* with rs56245262	LD* with rs6822503	1000 Genomes Frequency					
				Afffected child1	Afffected child2	Unafffected child1	Unafffected child2	Mother	Father					ALL	AFR	AMR	EAS	EUR	SAS
12650224	3'UTR	38825552	A	Hom	Hom	Het	-	Het	Het	Tier 2	0.0023	0.46	<0.2	0.21	0.33	0.17	0.28	0.03	0.20
6822503	3'UTR	38826076	A	Hom	Hom	Het	-	Het	Het		0.0082	<0.2	1	0.63	0.75	0.48	0.73	0.54	0.57
12645200	3'UTR	38826429	T	Hom	Hom	Het	-	Het	Het		0.0082	<0.2	0.97	0.63	0.75	0.48	0.73	0.54	0.57
5743826	3'UTR	38827855	T	Hom	Hom	Het	-	Het	Het		0.0029	<0.2	<0.2	0.04	0.09	0.02	0.00	0.02	0.06
3775073	Exon (p.Lys421Lys)	38829832	C	Hom	Hom	Het	-	Het	Het	NA	0.44	<0.2	0.83	0.46	0.74	0.32	0.40	0.31	0.39
3821985	Exon (p.Thr361Thr)	38830012	C	Hom	Hom	Het	-	Het	Het	NA	0.50	<0.2	0.58	0.44	0.69	0.31	0.40	0.31	0.39
35220466	Exon (p.Arg247Lys)	38830355	T	Het	Het	Het	-	-	Het	Tier 1	ND	<0.2	<0.2	0.006	0.021	0.001	0.000	0.000	0.000
5743809	Exon (p.Leu194Pro)	38830514	G	Het	Het	-	-	Het	-		ND	<0.2	<0.2	0.015	0.054	0.006	0.001	0.000	0.000
5743806	Intronic	38831767	G	Hom	Hom	Het	-	Het	Het	NA	0.16	<0.2	0.48	0.42	0.65	0.30	0.39	0.28	0.38
5743802	Intronic	38832168	C	Hom	Hom	Het	-	Het	Het	NA	0.16	<0.2	0.48	0.42	0.65	0.30	0.39	0.28	0.38
5743792	Intronic	38833022	G	Hom	Hom	Het	-	Het	Het	NA	0.16	0.52	<0.2	0.21	0.33	0.17	0.29	0.03	0.20
5743790	Intronic	38833154	C	Hom	Hom	Het	-	Het	Het	NA	0.16	<0.2	0.46	0.42	0.65	0.30	0.39	0.28	0.38
5743788	Intronic	38833207	G	Hom	Hom	Het	-	Het	Het	NA	0.089	0.24	0.71	0.61	0.71	0.46	0.73	0.51	0.56
6531669	Intronic	38834951	G	Hom	Hom	Het	-	Het	Het	NA	0.16	0.21	0.65	0.44	0.70	0.30	0.39	0.28	0.38
6531670	Intronic	38835022	C	Hom	Hom	Het	-	Het	Het	NA	0.16	0.21	0.65	0.44	0.70	0.30	0.39	0.28	0.38
11489107	Intronic	38835613	C	Hom	Hom	Het	-	Het	Het	NA	ND	0.24	0.67	0.61	0.71	0.46	0.73	0.51	0.56
57130162	Intronic	38838464	A	Hom	Hom	Het	-	Het	Het	NA	ND	0.22	0.38	0.39	0.60	0.29	0.38	0.28	0.33
73146532	Intronic	38839123	T	Hom	Hom	Het	-	Het	Het	NA	ND	0.67	<0.2	0.24	0.43	0.18	0.28	0.03	0.20
73146534	Intronic	38839445	A	Hom	Hom	Het	-	Het	Het	NA	ND	0.71	<0.2	0.24	0.42	0.18	0.28	0.03	0.20
6833914	Intronic	38840162	T	Hom	Hom	Het	-	Het	Het	NA	0.12	0.2	0.42	0.41	0.61	0.29	0.40	0.28	0.38
60970881	Intronic	38840969	T	Hom	Hom	Het	-	Het	Het	NA	ND	0.59	0.26	0.28	0.52	0.20	0.30	0.04	0.25
61198556	Intronic	38841072	C	Hom	Hom	Het	-	Het	Het	NA	0.035	0.55	0.27	0.28	0.52	0.20	0.30	0.04	0.25
58239725	Intronic	38841081	A	Hom	Hom	Het	-	Het	Het	NA	0.035	0.62	0.23	0.27	0.49	0.20	0.30	0.04	0.25
59295951	Intronic	38841108	G	Hom	Hom	Het	-	Het	Het	NA	0.033	0.53	0.25	0.28	0.52	0.20	0.30	0.04	0.25
12642845	Intronic	38841702	C	Hom	Hom	Het	-	Het	Het	NA	0.12	0.55	<0.2	0.22	0.32	0.22	0.30	0.03	0.20
73146554	Intronic	38845502	T	Hom	Hom	Het	-	Het	Het	NA	ND	0.94	<0.2	0.25	0.44	0.24	0.28	0.03	0.20
56245262	Intronic	38845609	A	Hom	Hom	Het	-	Het	Het	NA	6.9E-06	1	<0.2	0.41	0.45	0.40	0.55	0.26	0.38
73146558	Intronic	38846274	G	Hom	Hom	Het	-	Het	Het	NA	ND	0.94	<0.2	0.25	0.44	0.24	0.28	0.03	0.20
80230721	Intronic	38846293	G	Hom	Hom	Het	-	Het	Het	NA	ND	0.94	<0.2	0.25	0.44	0.24	0.28	0.03	0.20
56083757	Intronic	38846345	G	Hom	Hom	Het	-	Het	Het	NA	6.9E-06	1	<0.2	0.41	0.45	0.40	0.55	0.26	0.38
7669329	Intronic	38846719	C	Hom	Hom	Het	-	Het	Het	NA	8.9E-06	1	<0.2	0.41	0.45	0.40	0.56	0.26	0.38
7685852	Intronic	38846885	A	Hom	Hom	Het	-	Het	Het	NA	0.086	0.93	<0.2	0.25	0.44	0.24	0.28	0.03	0.20
6848545	Intronic	38850710	G	Hom	Hom	Het	-	Het	Het	NA	ND	0.89	<0.2	0.25	0.44	0.24	0.28	0.03	0.20
56060734	Intronic	38851691	A	Hom	Hom	Het	-	Het	Het	NA	ND	<0.2	<0.2	0.41	0.45	0.40	0.56	0.26	0.38
9684929	Intronic	38852387	T	Hom	Hom	Het	-	Het	Het	NA	ND	<0.2	<0.2	0.25	0.44	0.24	0.28	0.03	0.20
9685553	Intronic	38852689	A	Hom	Hom	Het	-	Het	Het	NA	ND	<0.2	<0.2	0.25	0.44	0.24	0.28	0.03	0.20
148906978	Intronic	38854369	T	Hom	Hom	Het	-	Het	Het	NA	ND	0.61	<0.2	0.41	0.46	0.40	0.56	0.26	0.38
6833997	Intronic	38855491	T	Hom	Hom	Het	-	Het	Het	NA	0.10	0.9	<0.2	0.25	0.44	0.24	0.28	0.03	0.20
7665515	Intronic	38856403	C	Hom	Hom	Het	-	Het	Het	NA	0.10	0.91	<0.2	0.25	0.44	0.24	0.28	0.03	0.20
7681879	Intronic	38856553	A	Hom	Hom	Het	-	Het	Het	NA	0.10	0.91	<0.2	0.25	0.44	0.24	0.28	0.03	0.21
6837101	Promoter	38858889	A	Hom	Hom	Het	-	Het	Het	Tier 2	0.03	0.51	0.25	0.38	0.57	0.34	0.35	0.29	0.31

Hom: homozygous of risk allele, Het: heterozygous of risk allele, -: homozygous of non-risk allele, ND: no data, NA: not applicable

*: LD r^2 in African in Haploreg
